# Antimicrobial Residues in Poultry Litter: Assessing the Association of Antimicrobial Persistence with Resistant *Escherichia coli* Strains

**DOI:** 10.3390/antibiotics14010089

**Published:** 2025-01-14

**Authors:** Paula Cortés, Ekaterina Pokrant, Karina Yévenes, Aldo Maddaleno, Andrés Flores, María Belén Vargas, Lina Trincado, Matías Maturana, Lisette Lapierre, Javiera Cornejo

**Affiliations:** 1 Laboratory of Veterinary Pharmacology (FARMAVET), Faculty of Veterinary and Animal Sciences, University of Chile, Avenida Santa Rosa 11735, La Pintana, Santiago 8820808, Chile; paula.cortes@ug.uchile.cl (P.C.); katiavalerievna@uchile.cl (E.P.); amaddaleno@veterinaria.uchile.cl (A.M.); andres.flores@veterinaria.uchile.cl (A.F.); lina.trincado@veterinaria.uchile.cl (L.T.); matias.maturana@ug.uchile.cl (M.M.); 2 Doctorate Program of Forestry, Agriculture, and Veterinary Sciences (DCSAV), University of Chile, Av. Santa Rosa 11315, La Pintana, Santiago 8820808, Chile; kyevenes@ug.uchile.cl (K.Y.); maria.vargas.s@ug.uchile.cl (M.B.V.); 3 Laboratory of Food Safety, Department of Preventive Animal Medicine, Faculty of Veterinary and Animal Sciences, University of Chile, Avenida Santa Rosa 11735, La Pintana, Santiago 8820808, Chile; llapierre@uchile.cl

**Keywords:** antimicrobial resistance, antimicrobial residues, persistence, *Escherichia coli*, sulfachloropyridazine, oxytetracycline, enrofloxacin, UPLC-MS/MS, chicken litter

## Abstract

**Objective**: We set out to evaluate the persistence of sulfachloropyridazine, oxytetracycline, and enrofloxacin in broiler chicken litter following therapeutic oral treatment and its association with the isolation of *Escherichia coli* resistant to these antimicrobials. **Methods**: Forty broiler chickens were raised under controlled conditions and divided into three experimental groups, each treated with a different antimicrobial, in addition to an untreated/control group. Litter samples were collected post treatment, analyzed by UPLC-MS/MS, and processed for the isolation of *E. coli*. The antimicrobial susceptibility of *E. coli* was assessed using the Kirby–Bauer disk diffusion method. **Results**: Chemical analysis detected concentrations of antimicrobials throughout post treatment, reaching maxima of 42,910.14 μg kg^−1^, 92,712 μg kg^−1^, and 9567 μg kg^−1^ for sulfachloropyridazine, oxytetracycline plus 4-epi-oxytetracycline, and enrofloxacin plus ciprofloxacin, respectively. It was estimated that the concentrations of sulfachloropyridazine, oxytetracycline, and enrofloxacin would persist in broiler litter for 61, 244, and 514 days, respectively. A very strong association was observed between the presence of antimicrobial residues and the antimicrobial resistance of *E. coli* (*p*-value < 0.0001, and Cramer’s coefficient of 0.47), and an independence between the level of residue concentration and susceptibility (*p*-value 0.5262). **Conclusions**: The persistence of antimicrobial residues contributes to the selection of resistant bacteria, regardless of persistent antimicrobial concentrations. These findings highlight the need for stricter regulations on poultry litter management, including residue thresholds and resistance monitoring, to minimize environmental and public health risks. Proper treatment of poultry litter is essential to ensure its sustainable and safe re-use in agricultural systems.

## 1. Introduction

Antimicrobials are essential in poultry production for treating birds affected by infectious diseases. Among these, fluoroquinolones, tetracyclines, and sulfonamides are critically important veterinary antimicrobial families used in the poultry industry [1]. This is relevant because it has been reported that birds can excrete up to 90% of these as either the original compound or as a metabolite, accumulating in the litter and persisting for variable periods [2,3,4,5,6].

The persistence of antimicrobial residues in poultry litter is significant, as each bird generates between 1.5 and 5.7 kg of litter over the course of a 42-day production cycle. Furthermore, poultry litter is used to fertilize agricultural soils and supplement animal diets due to its chemical and nutritional composition [7,8,9]. As a result of their re-use, these residues can spread to soils, water, and crops intended for human consumption [10].

Various studies indicate that the use of poultry litter poses risks to human, environmental, and animal health. For example, Hubbard et al. [11] concluded that its application as an agricultural fertilizer leads to contamination of groundwater and surface water with pathogenic bacteria and chemical residues, posing a risk to animals and humans who consume them. Furthermore, Ngogang et al. [12] found that poultry litter can serve as a source of multidrug-resistant *E. coli*. Similarly, Fatoba et al. [13] isolated *E. coli* strains before and after the application of poultry litter to agricultural soil and found that the highest number of resistant isolates occurred in fertilized soil (71.9%), followed by poultry litter (27.1%), and soil samples collected before litter application (1%). In addition, Zhao et al. [14] detected residues of tetracyclines, quinolones, sulfonamides, and macrolides in peanut plants grown in soils fertilized with animal waste. The authors indicated that consuming crops with antimicrobial residues is risky for humans, either at high doses or after prolonged exposure, due to potential side effects on children’s growth and hematopoietic function, as well as the human microbiome.

Recent studies have detected concentrations of antimicrobial residues in poultry litter components following therapeutic use [4,6]. Other studies support these findings and even estimated the persistence of antimicrobial residues in poultry litter components. In broiler droppings, laboratory studies have determined that the time required for 90% degradation of enrofloxacin (EFX) is 343 days, for oxytetracycline (OTC) 221 days, and for sulfachloropyridazine (SCP) 38 days [2]. In feathers, antimicrobial concentrations have been estimated to persist after treatment for at least 24 days for EFX, 38 days for SCP, and 45 days for OTC [3,15,16].

The presence of sub-MIC (minimum inhibitory concentration) levels of antibiotics in the environment leads to the selection and co-selection of resistant bacteria, favoring the persistence of resistant strains [17]. Recent studies suggest that antibiotic resistance can be selected due to the antibiotics present during poultry production [18]. One of the most studied bacteria as an indicator of antimicrobial resistance (AMR) is *E. coli*, which is part of the intestinal microbiota of birds; depending on whether virulence factors are present, this bacterium may be associated with the occurrence of intestinal or extraintestinal diseases. AMR studies in avian pathogens have described the resistance rate of *E. coli* as having increased over time, which may be promoted by its genomic plasticity, thereby facilitating the acquisition and transmission of resistance genes [19,20].

Despite the widespread re-use of poultry litter, there is limited information on the behavior of antimicrobial residues commonly used in the poultry industry within broiler litter under production-like conditions following complete ingestion, as well as their impact on bacterial resistance. This study addresses these knowledge gaps and aligns with international research priorities, such as those established by the European Union, which emphasize evaluating the long-term effects of antimicrobial use in animals on public health and environmental sustainability [21,22]. Specifically, it assessed the persistence of OTC, EFX, and SCP residues in broiler chicken litter post treatment and their association with the isolation of resistant *Escherichia coli*. This work provides key insights into the persistence of antimicrobials in untreated broiler litter and their role in selecting resistant bacteria, supporting the development of safer management strategies and promoting sustainable use within the circular economy.

## 2. Results

### 2.1. Verification of the Analytical Method

The results of the analytical method verification indicated that the method was highly linear (R^2^ ≥ 0.99) and sensitive for the detection and quantification of the studied antimicrobials and their main residues. The detection limits were at least 2.50 μg kg^−1^ and quantification limits were at least 3.90 μg kg^−1^. These results are detailed in Table 1.

In addition, the method met the specificity parameter, as no interference was detected at the analyte retention time when comparing pure drug injections with 20 blank samples.

### 2.2. Detection and Quantification of Antimicrobials in Broiler Litter

**Figure 1:**
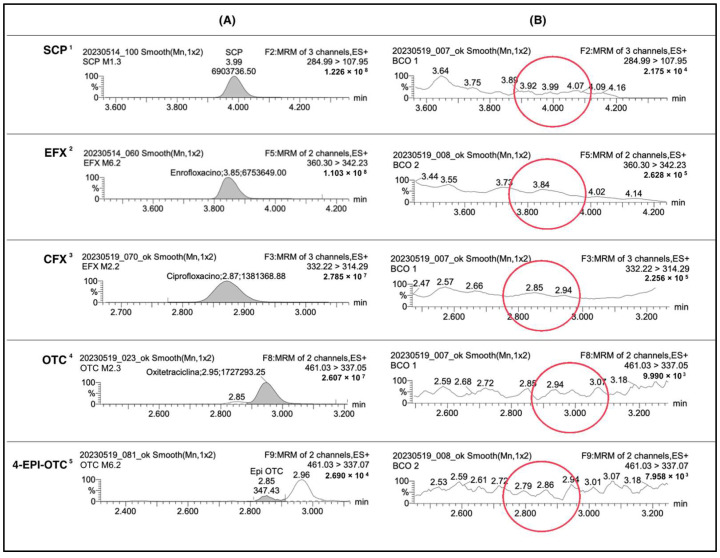
Chromatograms of positive sample injections of each analyte (Column (**A**)), and blank samples (Column (**B**)). The peak present in the positive samples is not present in the blank samples (red circles). SCP ^1^: sulfachloropyridazine; EFX ^2^: enrofloxacin; CFX ^3^: ciprofloxacin; OTC ^4^: oxytetracycline; 4-EPI-OTC ^5^: 4-epi-oxytetracycline.

Samples were analyzed in triplicate to assess the precision of the analytical result and to ensure its validity. Figure 1 presents examples of chromatograms from positive and blank samples from the experiment for comparison.

Concentrations of sulfachloropyridazine (SCP) in litter from treated birds ranged from 207 to 42,910 μg kg^−1^ post treatment. The concentrations of oxytetracycline (OTC) combined with 4-epi-oxytetracycline in the litter of treated animals varied between 25,111 and 92,712 μg kg^−1^. The concentrations of enrofloxacin (EFX) along with its active metabolite varied from 8.79 to 9567 μg kg^−1^. Table 2 details the results of the triplicate analysis of the litter and average concentrations of all analytes studied post treatment.

### 2.3. Determination of Antimicrobial Residue Persistence in Broiler Litter

**Figure 2:**
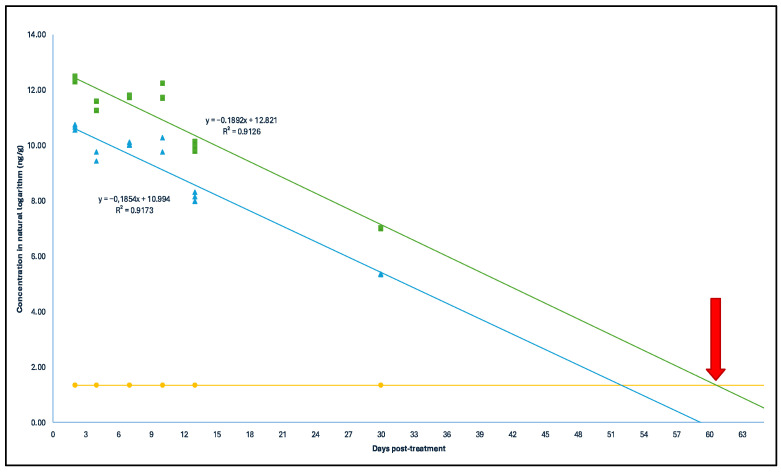
Depletion curve of SCP concentrations (in natural logarithm) post treatment (days) in the treated broiler litter. Green squares: concentration in natural logarithm with a 95% confidence level; blue triangles: concentration in natural logarithm; yellow spheres: limit of quantification of SCP in the analytical method (3.9 μg kg^−1^). Red arrow: Depletion time of Sulfachloropyridazine time = 61 days.

**Figure 3:**
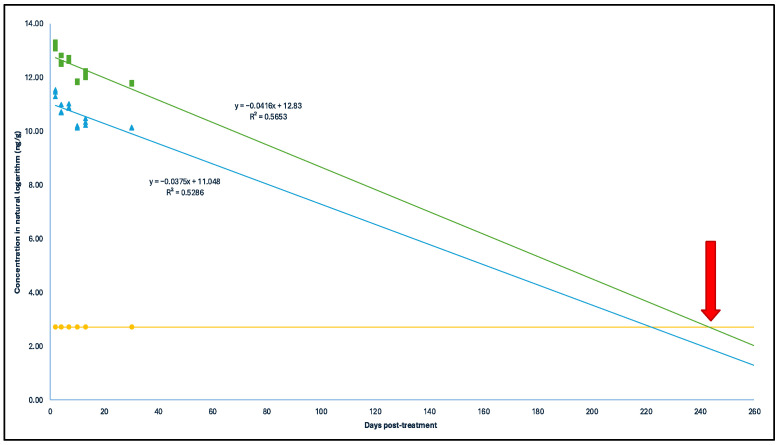
Depletion curve of OTC and 4-epi-oxytetracycline concentration (in natural logarithm) post treatment (days) in the treated broiler litter. Green squares: concentration in natural logarithm with a 95% confidence level; blue triangles: concentration in natural logarithm; yellow spheres: limit of quantification of OTC in the analytical method (15 μg kg^−1^). Red arrow: Depletion time of Oxytetracycline time = 244 days.

**Figure 4:**
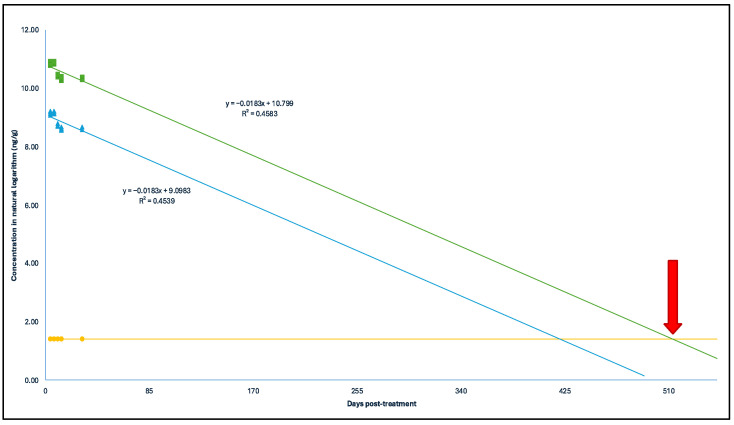
Depletion curve of EFX and ciprofloxacin concentrations (in natural logarithm) post treatment (days) in the treated broiler litter. Green squares: concentration in natural logarithm with a 95% confidence level; blue triangles: concentration in natural logarithm; yellow spheres: limit of quantification of EFX in the analytical method (4.1 μg kg^−1^). Red arrow: Depletion time of Enrofloxacin time = 514 days.

The concentrations of each antimicrobial post treatment were projected over time to statistically determine the persistence of the different compounds. With a 95% confidence level, it was estimated that the concentrations of SCP would be equal to or less than the limit of quantification (LOQ) of the analytical method in broiler litter by day 61 post treatment (Figure 2). In the case of OTC, this would occur by day 244 (Figure 3), and for EFX, by day 514 (Figure 4). It is worth noting that, for the analysis of this last antimicrobial, the first sampling was not considered due to significantly lower concentrations.

### 2.4. Antimicrobial Resistant E. coli Strains

From the processed litter samples, 10 strains of *E. coli* were isolated from each sample: 5 strains from the control group and 5 strains from the treatment group. In total, 90 strains were isolated post treatment, with species confirmation performed by identifying the *uspA* gene using PCR.

**Figure 5:**
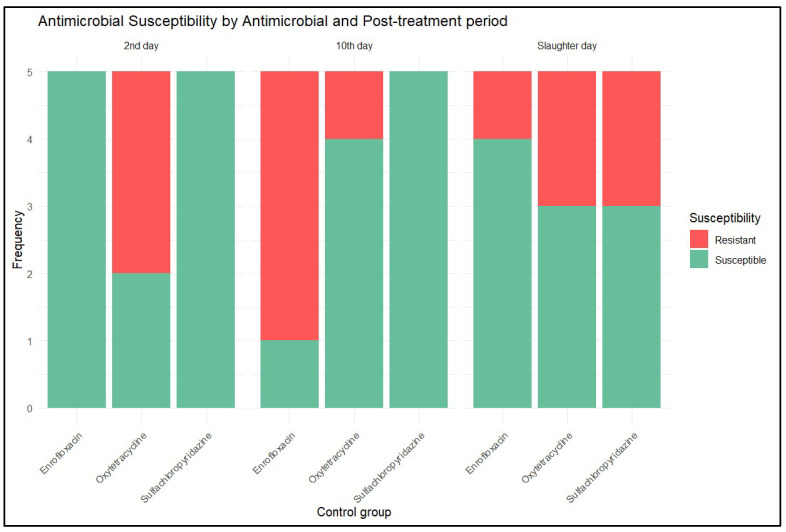
Frequency of *E. coli* strains isolated from the litter of the control group, characterized by phenotypic resistance to the studied antimicrobial as resistant (red) or susceptible (green), on days 2 and 10 post treatment, and on the slaughter day (corresponds to day 30 post treatment for SCP, day 15 post treatment for OTC, day 17 post treatment for EFX).

In the phenotypic antimicrobial susceptibility testing in the control group, only susceptible and resistant strains of *E. coli* were identified, with 71.1% of isolates being susceptible to the studied antimicrobials. Figure 5 details the results of the disk diffusion test on strains from the litter of the control group.

**Figure 6:**
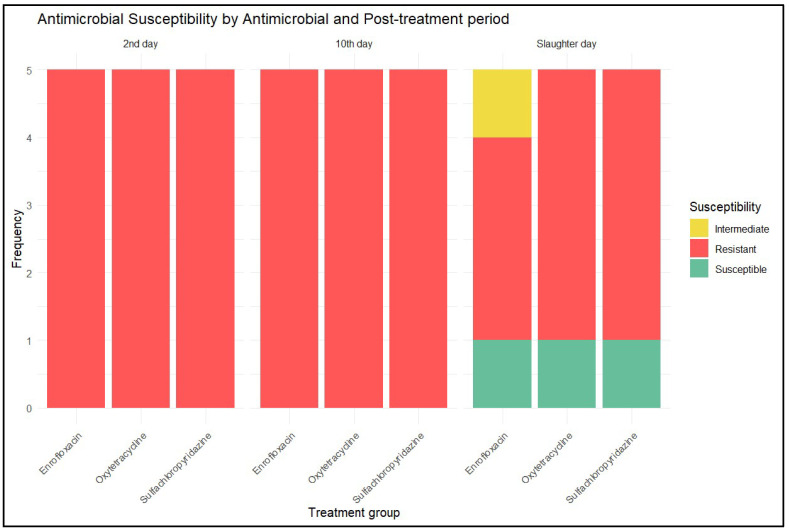
Frequencies of *E. coli* strains isolated from the litter of birds in the different treatment groups, characterized by phenotypic resistance to the studied antimicrobial as resistant (red), intermediate (yellow), and susceptible (green), corresponding to days 2 and 10 post treatment, and on the slaughter day (corresponds to day 30 post treatment for SCP, day 15 post treatment for OTC, and day 17 post treatment for EFX).

In the treated bird litter, susceptible, intermediate, and resistant strains of *E. coli* were found. The findings revealed that 93.3% of the isolated strains were resistant to the family of antimicrobials used to treat the chickens. Figure 6 details the phenotypic resistance characterization of the strains exposed to the antimicrobial used to treat the birds.

As shown in Figure 6, 100% of the *E. coli* strains were resistant to all three studied antimicrobials on days 2 and 10 post treatment. On the slaughter day, for the strains treated with SCP and OTC, 80% were resistant and 20% were susceptible. Strains from birds treated with EFX were 60% resistant, 20% susceptible, and 20% intermediate.

Table 3 shows a prevalence table comparing the percentages of resistant strains between the treatment group and the control group post treatment.

### 2.5. Statistical Analysis Results

The analysis using the non-parametric Chi-square test for independence, aimed at evaluating the association between the presence of antimicrobial residues in poultry litter and the susceptibility of *E. coli* strains, yielded a *p*-value of <0.0001. Since this value is less than the significance level (*p* < 0.05), there is evidence allowing us to reject the null hypothesis, establishing that there is a statistically significant relationship between the two variables. Specifically, the presence of antimicrobial residues in poultry litter is associated with resistant strains of *E. coli*. Additionally, Cramer’s V coefficient is 0.47, indicating that this association is very strong according to the criteria established by Akoglu [23].

When evaluating the association between the concentration levels of residues categorized as low and high and antimicrobial susceptibility, the *p*-value was 0.5262. Since this value is greater than the significance level, there is insufficient evidence to suggest that the antimicrobial susceptibility of *E. coli* strains isolated from the litter of treated chickens is associated with the concentration level of drug residues. In other words, there is no relationship between the concentration levels of antimicrobial residues persisting in poultry litter post treatment and the resistance of the isolated *E. coli* strains.

## 3. Discussion

The concentrations of SCP and OTC in the litter of treated chickens decreased over time post treatment. However, the initial average concentration of EFX in the litter of treated chickens was 8.79 µg kg^−1^, which increased to over 9000 µg kg^−1^ at the next sampling point. This result can be explained by the pharmacokinetics of this antimicrobial, characterized by low plasma protein binding and a high volume of distribution [24,25]. At the first sampling, it is possible that EFX was still in the distribution phase in the chickens’ bodies, resulting in reduced excretion 48 h post treatment, which subsequently increased to a maximum of 9567 µg kg^−1^ at 168 h post treatment.

The behavior of antimicrobial residues post treatment aligns with other studies that treated birds at therapeutic doses via orogastric catheterization, such as those in references [4,6], which evaluated SCP in chicken droppings, and [26], which analyzed OTC along with 4-epi-oxytetracycline. Moreover, the pattern of increasing concentrations of EFX in the initial days post treatment resembles findings from other studies conducted on bird droppings, such as Pokrant et al. [4] and Slana et al. [27]. However, Sureshkumar and Sarathchandra [28] observed initial concentrations close to 4000 µg kg^−1^, which decreased until day 9 post treatment, where no concentrations were detected.

EFX showed the longest estimated persistence, followed by OTC and SCP. This order aligned with the findings of the study conducted in the laboratory by Berendsen et al. [2], which established the DT_90_ for the same antimicrobials in poultry droppings. However, in vivo studies estimating the persistence of antimicrobials in droppings have tended to project shorter periods, reducing to 50 and 55 days for SCP and EFX, respectively [4]. The difference in persistence observed in poultry litter could be attributed to the accumulation of droppings in this by-product over the production cycle, which increases the concentration of residues and, therefore, leads to a longer projected persistence. Pokrant et al. [26] demonstrated differences in the concentrations in litter, which can initially be up to ten times higher.

Other studies have detected residues of the antimicrobials evaluated in this study in poultry litter. For instance, Vargas et al. [29] reported similar concentrations of SCP in poultry litter following therapeutic treatment, while Fučík et al. [30] detected up to 70,000 μg kg^−1^ of EFX during the initial days of treatment in water. Additionally, Pokrant et al. [26] found approximately 50,000 μg kg^−1^ of OTC in poultry litter within two weeks post treatment. These findings highlight the presence of these compounds in poultry waste after treatment and the variability in concentrations observed across studies due to factors such as experimental design, environmental conditions, and the number of animals involved.

To date, and to our knowledge, there are no publications estimating the persistence of SCP, OTC, or EFX in poultry litter. However, recent investigations have provided significant findings that suggest this phenomenon. Pokrant et al. [4] detected SCP concentrations in broiler droppings even four days after the withdrawal period established by the pharmaceutical formulation used. Similarly, another study in 2021 detected OTC concentrations in litter from orally treated chickens at therapeutic doses, which remained between 10,000 and 15,000 µg kg^−1^ for up to two weeks post slaughter [26]. These results are consistent with the projected persistence in the present study, where it was estimated that the antimicrobials would remain in the litter beyond the withdrawal period of the pharmaceutical formulation, and even beyond the productive cycle of the treated birds.

Throughout the study, 30.3% of the administered SCP dose (105,847.45 μg kg^−1^), 4.3% of OTC and its metabolite 4-EPI-OTC (280,962.71 μg kg^−1^), and 6.4% of EFX and its metabolite CFX (36,508.51 μg kg^−1^) were detected in broiler litter, relative to the total dose administered to the 10 chickens. Despite the lower concentrations in comparation to the total dose administered, these residues still pose significant environmental risks. The properties of antimicrobials play a key role in determining their behavior in soil when poultry litter is used as an agricultural fertilizer. Studies report that fluoroquinolones and tetracyclines possess characteristics that enable them to accumulate on the soil surface and persist due to interactions with other compounds, potentially affecting soil ecosystems and even crops. For instance, oxytetracycline exhibits a desorption rate of up to 2.3% in soils sampled from agricultural fields. In contrast, sulfonamides have low soil adsorption, and yet they pose a significant risk of leaching into groundwater due to their high mobility [31].

Microbiological analyses of the litter from the groups treated with antimicrobials were similar in all three pens. All *E. coli* strains isolated on days 2 and 10 post treatment were resistant, and 80% of the strains remained resistant on the last day of the birds’ production cycle. Additionally, the high prevalence of resistant strains observed after treatment, compared to groups not exposed to antimicrobials, is consistent with other studies, such as Burow et al. [32], who found that in fecal isolates from pigs treated with colistin, tetracyclines, beta-lactams, and macrolides, higher antimicrobial resistance rates could be observed compared to untreated pigs at various sampling points. Similarly, Pokrant et al. [18] found significant differences in the isolates from the litter of birds treated with OTC compared to untreated birds, confirming the effect of OTC on the resistance of *E. coli* strains to tetracyclines isolated from the litter. The increase in *E. coli* resistance rates could also be linked to the genomic plasticity of *E. coli* in response to stressors, such as the persistent antimicrobial residues in the litter. These residues, even at suboptimal concentrations, trigger intrinsic mechanisms and exert selective pressure on resistant strains, favoring their survival and proliferation and playing a fundamental role in the dynamics of antimicrobial resistance at the environmental level [33,34].

Resistant strains to the antibiotic discs were identified in the control group, even though these birds were housed in a separate room and were not exposed to the antimicrobial. This could be attributed to the composition of the intestinal microbiota in those birds. In previous research conducted by Xiong et al. [35] and Xu et al. [36], antimicrobial resistance genes were identified in the intestinal content and fresh droppings of broiler chickens, despite the fact that they had not been previously treated with antimicrobials.

It has also been suggested that the composition of the microbiota in chicken embryos is partly derived from the reproductive tract and cloaca of the hens [37,38]. Considering this, along with the fact that antimicrobials are widely used in the poultry industry for various purposes, it is possible that the experimental birds originated from breeder hens that had been previously treated with the studied antimicrobials. This could have contributed to the selective pressure for resistant bacteria in their microbiota, potentially transmitted through the eggs to the birds in this study. Studies such as Jansen et al. [39] have demonstrated that antibiotics, including EFX, doxycycline, and SCP, can be vertically transmitted from the mother hen to the broiler chickens through the egg and its shell, potentially contributing to the selective pressure for resistant bacteria.

The results of the statistical analyses are consistent with the literature, which describes that bacterial stressors such as antimicrobials can promote the acquisition of resistance genes and induce genetic mutations, leading to reduced susceptibility to the drug. Moreover, it is suggested that the use of antimicrobials, even at sub-inhibitory concentrations, generates an impact favoring the development of antimicrobial resistance in bacteria [40,41]. These findings also align with experimental trials conducted by researchers such as van der Horst et al. [42] and Chantziaras et al. [43], who demonstrated a high prevalence of resistant *E. coli* strains isolated from the droppings of chickens treated with antimicrobials at therapeutic, sub-therapeutic, and even higher than therapeutic doses. Additionally, the observed association between concentration levels and susceptibility can be attributed to the complexity of the poultry litter matrix. Factors such as pH, humidity, and water activity may influence bacterial fitness, potentially leading to the loss of resistance genes and, consequently, their expression [44,45].

According to the results obtained in this study, the risk of antimicrobial residue dissemination from poultry litter into the environment is considerable, especially if it is applied fresh. However, it has been reported that common treatments can degrade OTC, SCP, and EFX residues by nearly 100%. Specifically, degradation rates for OTC range from 97.2% to 99.8%, for SCP from 91% to 100%, and for EFX up to 99.96% [46,47]. Despite these degradation rates, it is important to note that components associated with bacterial resistance are not completely eliminated, such as antimicrobial resistance genes and genetic elements. Studies indicate that typical treatments applied to litter for its re-use in animal or agricultural production only achieve partial removal of these genetic components [48,49,50,51]. For example, da Silva Gonçalves [52] detected antimicrobial resistance genes in both fresh and treated poultry litter samples, indicating that bacterial populations can retain genetic material linked to resistance even after processing. This is particularly concerning, as it has been demonstrated that bacteria present in poultry litter can develop resistance through the acquisition of these genetic elements [53]. These findings emphasize the need for more effective litter management protocols that not only reduce antimicrobial residues but also target resistant bacteria and associated genetic components.

Currently, there are regulations and guidelines concerning the re-use of animal manure, including litter, for subsequent production cycles or agricultural applications based on soil nutrient requirements or gas emissions, and even for animal feed in different countries [54,55,56,57]. In the European Union [56,57], its direct use is prohibited for feeding and agricultural applications, requiring standard processing that includes parameters such as temperature, pressure, time, and particle size to reduce the risks of disease propagation and antimicrobial resistance. These regulations specify the colony-forming unit (CFU) limits for *Salmonella* spp. and *E. coli* after processing [58]. However, they do not address their antimicrobial susceptibility, resistance components, or permissible concentrations of antimicrobials in poultry litter. This gap is concerning, especially in light of the results obtained in this study and the associated risks, particularly if contaminants persist despite prior treatments.

The results of this study demonstrate the persistence of antimicrobial residues and the impact on microorganisms present in by-products of the poultry industry that are re-used for agricultural and livestock-feeding purposes. This situation raises significant concerns from a One Health perspective, as these risks may spread to the environment along with resistant bacteria, which could eventually re-enter the food chain.

## 4. Materials and Methods

### 4.1. Experimental Design

#### 4.1.1. Experimental Animals

Forty Ross 308 male broiler chickens (Ross^®^ Aviagen Inc., Huntsville, AL, USA) were sourced from a large-scale local commercial supplier and reared from day one of life in 1 m^2^ pens over a 42-day production cycle. The environmental conditions, including light, temperature (maintained at 20–30 °C), humidity (ranging from 50 to 60%), and ventilation, were maintained following the breed’s management guidelines [59]. Additionally, the birds had free access to water and feed, with an initial diet transitioning to a final phase at 21 days of life.

The rearing conditions and practices, animal welfare, and slaughter of the birds were approved by the Institutional Animal Care and Use Committee of the University of Chile (Certificate No. 21471—VET—UCH). The biosecurity measures throughout the study were sanctioned by the Biosecurity Committee of the Faculty of Veterinary and Animal Sciences (Certificate No. 180). The handling and care protocol for the birds was based on Law No. 20.380 “On the Protection of Animals” [60] and Directive 2010/63/EU on the protection of animals used for scientific purposes [61]. The slaughter of the birds was carried out in accordance with the guidelines set forth by the American Veterinary Medical Association [62].

The experimental trial consisted of four experimental groups, each comprising 10 individuals selected at random. The birds were housed in pens with 10 cm of wood shavings, following the recommendations outlined in the “Poultry Industry Manual “ from the United States Department of Agriculture [63]. Based on these guidelines, the number of birds was calculated to accommodate 10 per pen, considering a maximum weight of 3.20 kg per bird and a density of 34.37 kg/m^2^. To prevent cross-contamination, the control group was kept in a separate room from the three treatment groups.

Both the wood shavings from litter and the feed were analyzed to rule out the presence of residues of the antimicrobials of interest.

#### 4.1.2. Treatment Protocol

The treatment of the birds varied between the pens and was orally administered by orogastric catheter to ensure complete ingestion of the antimicrobial. Thus, a different antimicrobial was administered to each pen, adhering to the withdrawal time of the pharmaceutical formulations used: 5 days for OTC, 10 days for EFX, and 30 days for SCP. The protocol consisted of a daily therapeutic dose of 59 mg kg^−1^ of OTC for 7 days, 10 mg kg^−1^ of EFX for 5 days, and 30 mg kg^−1^ of SCP for 5 days, for the corresponding group. The control group was administered distilled water.

#### 4.1.3. Sampling

Systematic grid sampling of poultry litter was carried out, according to IAEA guidelines [64], collecting 31 g for microbiological analysis and 18 g for chemical analysis per sampling point. Six sampling points were used for chemical analysis, where the first two points were every 48 h, the next three every 36 h, and the last one on the day of slaughter of the birds. For microbiological analysis, there were three sampling points, where a sample was taken on the second and tenth post-treatment days and on the slaughter day.

### 4.2. Antimicrobial Detection in Broiler Litter (UPLC-MS/MS)

#### 4.2.1. Chemicals and Reagents

HPLC-grade solvents were used to carry out the chemical extraction of the analytes of interest, such as water, acetonitrile, methanol, and an EDTA-McIlvaine buffer (0.1 M, pH 4.0 ± 0.1). EDTA-McIlvaine buffer preparation was based on a solution of 0.1 M citric acid and 0.2 M disodium phosphate in HPLC water. All solvents were sourced from Merck (Darmstadt, Germany), Fisher (Thermo Fisher Scientific, Waltham, MA, USA), or similar.

For chromatographic analysis, phase A consisting of 2.0 mM ammonium formate solution with 0.16% formic acid in water was used, while phase B consisted of 2.0 mM ammonium formate with 0.16% formic acid in methanol.

#### 4.2.2. Standards and Working Solutions

For the detection and quantification of residues in the experimental samples, certified standards of oxytetracycline (OTC), 4-epi-oxytetracycline (4-epi-OTC), enrofloxacin (EFX), ciprofloxacin (CFX), and sulfachloropyridazine (SCP), all with a purity higher than 90%, were obtained from Sigma Aldrich, Inc. (Merck KGaA, Darmstadt, Germany). Additionally, internal standards including enrofloxacin-D5 (EFXD5), sulfamethazine-phenyl-13C6 (SMZ 13C6), and tetracycline-D6 (TC-D6) were sourced from Toronto Research Chemicals (Toronto, ON, Canada).

The stock solution of each standard was prepared at a concentration of 1000 µg/mL in methanol. From these stock solutions, two intermediate or working solutions were prepared. One solution contained a mixture of all the antimicrobials (SCP, EFX, CFX, OTC, and 4-epi-OTC), while the other comprised the internal standards (SMZ 13C6, EFX-D5, and TC-D6). Both intermediate solutions were prepared at a concentration of 1000 ng/mL in methanol.

#### 4.2.3. Chemical Analysis of Samples

The litter samples were ground, homogenized, and then weighed (1 ± 0.01 g) before fortification with the internal standard mix. Solid–liquid extraction was then performed following the protocol of Yévenes et al. [65]. The extraction of analytes began with the addition of 8 mL of EDTA-McIlvaine buffer (0.1 M, pH 4.0) and 2 mL of acetonitrile to the samples, followed by shaking, sonication, and centrifugation. The supernatant was filtered through 150 mm GF/A-grade glass microfiber Whatman^TM^ filters (Merck, Darmstadt, Germany), diluted, and passed through OASIS^®^HLB solid-phase extraction columns (6 cc, 200 mg sorbent per cartridge, 30 µm, 30/pk; Waters™, Milford, CT, USA). After washing with HPLC water and drying under a vacuum, samples were eluted with 10 mL methanol and evaporated under a nitrogen flow. The samples were then reconstituted and transferred to vials using syringe filters (13 mm × 0.22 µm; Millex^®^, Merck KGaA, Burlington, MA, USA).

Subsequently, the analytes were detected using the ACQUITY UPLC^®^ I-Class System (Waters™, Milford, MA, USA), coupled with a Xevo TQ-S micro triple quadrupole mass spectrometer (Waters™, Milford, MA, USA). For this analysis, an ACQUITY UPLC^®^ HSS T3 analytical column (1.8 µm, 2.1 × 100 mm; Waters™ Corp, USA) was used. The samples were integrated using MassLynx™ software, version 4.2.

The analytical methodology was previously verified. This verification involved the assessment of retention time, linearity, specificity, limit of detection (LOD), and limit of quantification (LOQ), based on international standards such as EU Guideline 808/2021, VICH GL 49, and VICH GL 2 [66,67,68]. Acceptance criteria were established as follows: the relative standard deviation (RSD) of the retention time for each analyte should be ≤1%; for linearity, an R^2^ ≥ 0.95 with an RSD ≤ 25% was evaluated, using concentrations of 0, 2.5, 5, 10, 15, 30, 60, and 120 µg kg^−1^, with the first fortified point of the curve adjusted according to the detection ranges of each analyte; for specificity, 20 blank samples should show no interference with the retention time of the analyte; and finally, for the limits of detection and quantification, the signal-to-noise ratios should be at minima of 3:1 and 10:1, respectively.

To confirm the presence of the analytes, the retention time corresponding to each analyte was evaluated. Additionally, a relative retention time of ±2.5% was considered, along with an ion ratio with a relative deviation of ±40%, and a minimum signal-to-noise ratio of 3:1.

### 4.3. Persistence Determination

The analytes were quantified using linear regression analysis of a calibration curve constructed in a blank matrix, with a minimum coefficient of determination (R^2^) of 0.95. To determine the day on which the concentration of each antimicrobial was equal to or below the LOQ in the broiler litter matrix, EMA [69] recommendations were followed, and the Microsoft^®^ Excel tool for Mac Version 16.92 (24120731) was used. The antimicrobial concentrations were converted to a logarithmic scale, and a linear regression analysis was then performed using concentration and time as the variables. Concentration values obtained via LC-MS/MS were log-transformed for a better statistical fit, and a 95% confidence level was applied. To estimate the depletion time of antimicrobial residues in the matrix, the withdrawal periods were defined as the point where the 95% upper tolerance limit fell below the limit of quantification (LOQ).

### 4.4. Isolation, Confirmation, and Susceptibility Testing of Strains

For *E. coli* isolation, the litter samples were pre-enriched in 225 mL of buffered peptone water (BPW) broth (Liofil-chem™, Roseto degli Abruzzi, Italy), then plated on MacConkey agar (BD Difco™, Sparks, MD, USA), and incubated at 37 °C for 24 h. After incubation, five characteristic *E. coli* colonies were selected and confirmed through identification of the *uspA* gene [70].

The detection of the *uspA* gene was performed using conventional polymerase chain reaction (PCR) techniques. DNA extraction was carried out using the boiling method. The isolates stored in TSB + 20% glycerol were plated on MacConkey agar (BD Difco™) and incubated at 37 °C for 18 to 24 h. They were then re-plated on TSA (Liofil-chem™, Roseto degli Abruzzi, Italy) and incubated under the same conditions. A loopful of bacterial growth was suspended in a 1.5 mL polypropylene tube with sterile saline, which was then homogenized and centrifuged for 3 min. The supernatant was discarded, and 1 mL of sterile saline was added to each tube for re-homogenization. Subsequently, the samples were subjected to a temperature of 100 °C in a Thermoblock (ACCUBLOCK™, Labnet International, Cary, NC, USA) for 10 min. Finally, the tubes were centrifuged for 5 min in a D1524R microcentrifuge (DLAB^®^, Beijing, China), and 400 μL of the supernatant was stored in Eppendorf^®^ tubes (Eppendorf, Hamburg, Germany) at −20 °C for further analysis.

The PCR was conducted using a Gene Explorer Model GE-48DS thermocycler (BIO-ERTM^®^, Hangzhou, China). The protocol began with an initial denaturation phase at 94 °C for 5 min, followed by 25 cycles consisting of denaturation at 94 °C for 30 s, annealing at 58 °C for 30 s, and elongation at 72 °C for 40 s, ending with a final extension at 72 °C for 5 min [70].

The PCR products were stained with SafeView^®^ Plus (Fermelo Biotec, Santiago, Chile) and visualized by agarose gel electrophoresis (1.5% (*w*/*v*) agarose in 1× TAE buffer). The amplicons were visualized using a UV transilluminator (SmartBlue™, Accuris Instruments, Edison, NJ, USA), and the size of the PCR products was determined using a 100 base pair (bp) molecular marker (Maestrogen, Hsinchu, Taiwan).

The confirmed *E. coli* isolates were transferred to tryptic soy broth and incubated at 37 °C for 18–24 h. Subsequently, the bacterial suspension was diluted to an optical density equivalent to 0.5 McFarland (1.5 × 10^8^ CFU/mL), as determined using a HALO RB-10 spectrophotometer (Dynamica GmbH, Salzburg, Austria) (absorbance at 625 nm). Next, the sample was plated on a Petri dish with Mueller–Hinton agar (Sigma Aldrich^®^, Saint Louis, MO, USA), and a susceptibility test disk of enrofloxacin, tetracycline, and sulfamethoxazole–trimethoprim (OXOID^®^, Hants, UK) was placed according to the treatment given to the birds. The plates were then incubated for 16–18 h at 35 ± 2 °C. After incubation, the inhibition zone was measured and classified as “Resistant”, “Susceptible”, or “Intermediate” according to the official interpretation manuals of the Clinical and Laboratory Standards Institute [71,72].

The positive control used was *E. coli* ATCC 10536, while *E. coli* ATCC 25922 was used as the quality control.

### 4.5. Statistical Analysis

To determine the association between concentration and susceptibility variables, two non-parametric Chi-square tests (95% confidence level, *p*-value of 0.05) were conducted. The first analysis evaluated the presence of antimicrobials and susceptibility, while the second analyzed the association between high and low concentration levels (above and below the maximum residue limit of each antimicrobial in chicken muscle according to the European Union [73,74,75], which is 100 µg kg^−1^ for all studied antimicrobials) and susceptibility. Strains were classified as susceptible or non-susceptible, grouping resistant and intermediate strains into the latter category. Additionally, Cramer’s coefficient was calculated to evaluate the strength of the association between the variables. The association between the variables was conducted using the InfoStat^®^ statistical software, version 2020I.

## 5. Conclusions

Antimicrobials administered orally via orogastric catheter at therapeutic doses in broiler chickens can persist for prolonged periods in the litter of these animals, even longer than the withdrawal period established for the pharmaceutical formulation of the antimicrobial.

The presence of residues of EFX, OTC, and SCP in the litter of treated broiler chickens is associated with a higher prevalence of antimicrobial-resistant *E. coli* strains, regardless of the concentration of these antimicrobials. Consequently, the persistence of antimicrobials contributes to the development of resistant bacteria in the environment. This emphasizes the need for stricter regulations on processing protocols for poultry litter before re-use, with residue thresholds and susceptibility monitoring strategies to mitigate risks to animals, humans, and the environment.

Finally, studies are recommended to investigate the treatment of poultry litter that could effectively eliminate the risk of spreading residues of veterinary medicines with antimicrobial activity and genetic components of bacterial resistance. Additionally, it is crucial to examine the mechanisms behind the persistence of resistance genes and to conduct long-term monitoring of residue degradation. Moreover, exploring the dynamics of microbiota at the individual level and the interactions of environmental microorganisms before, during, and after treatments using advanced techniques such as metagenomics could provide deeper insights. Such studies would enable the development of targeted strategies to mitigate these risks, ensuring the sustainable use of this poultry by-product without compromising public, animal, and environmental health.

## Figures and Tables

**Table 1:** Parameters of the multi-residual antimicrobial method for LC-MS/MS analysis in a poultry litter.

Analyte	Retention Time (Minutes)	LOD ^1^ (μg kg^−1^)	LOQ ^2^ (μg kg^−1^)
Oxitetracycline	2.96	5	15
4-epi-oxitetracycline	2.86	10	15
Enrofloxacin	3.84	2.50	4.10
Ciprofloxacin	3.85	2.50	4.80
Sulfachloropyridazine	3.97	2.50	3.90

^1^ LOD: limit of detection of analytical method; ^2^ LOQ: limit of quantification of analytical method.

**Table 2:** Concentrations of sulfachloropyridazine, oxytetracycline plus 4-epi-oxytetracycline, and enrofloxacin plus ciprofloxacin in the litter of treated broilers.

Day Post Treatment	Sulfachloropyridazine		Oxytetracycline + 4-Epi-oxytetracycline		Enrofloxacin + Ciprofloxacin	
	Average Concentration (μg kg^−1^) ± SD ^a^	RSD (%) ^b^	Average Concentration (μg kg^−1^) ± SD	RSD (%)	Average Concentration (μg kg^−1^) ± SD	RSD (%)
2	42,910.14 ± 4059.00	9.45	92,711.98 ± 11,154.29	12.03	8.79 ± 0.85	9.61
4	14,152.54 ± 2814.84	19.89	49,840.30 ± 9070.00	18.20	9424.79 ± 584.02	6.20
7	23,595.14 ± 1316.48	5.58	55,983.86 ± 4720.98	8.43	9566.58 ± 471.82	4.93
10	21,469.28 ± 7049.86	32.84	25,651.97 ± 868.66	3.39	6249.15 ± 278.49	4.46
13	3513.16 ± 611.24	17.40	31,663.96 ± 3918.62	12.38	5519.60 ± 371.92	6.74
Slaughter day ^c^	207.19 ± 12.22	5.90	25,110.64 ± 489.91	1.95	5739.60 ± 305.47	5.32

^a^ SD: standard deviation; ^b^ RSD%: relative standard deviation of triplicate samples, expressed as a percentage; ^c^ slaughter day corresponds to day 30 post treatment for SCP, day 15 post treatment for OTC, and day 17 post treatment for EFX.

**Table 3:** Percentages of *E. coli* strains resistant to the respective antimicrobial post treatment for the treatment group and the control group with a total of 15 *E. coli* strains isolated from each group per sample.

Day Post Treatment	Resistant Strains			
	Treatment Group		Control Group	
	Number of Strains	Prevalence (%)	Number of Strains	Prevalence (%)
2	15	100	3	20
10	15	100	5	33.33
Slaughter day *	12	80	5	33.33

* Slaughter day: day 42 of life for all birds, corresponding to day 30 post treatment for SCP, day 15 post treatment for OTC, and day 17 post treatment for EFX.

## Data Availability

Data are contained within the article.
